# Synchrotron Radiation‐Based In Situ GIWAXS for Metal Halide Perovskite Solution Spin‐Coating Fabrication

**DOI:** 10.1002/advs.202403778

**Published:** 2024-07-11

**Authors:** Yingguo Yang, Shanglei Feng, Xiaoxi Li, Minchao Qin, Lina Li, Xuyong Yang, Renzhong Tai

**Affiliations:** ^1^ School of Microelectronics Fudan University Shanghai 200433 China; ^2^ Shanghai Synchrotron Radiation Facility (SSRF) Shanghai Advanced Research Institute & Shanghai Institute of Applied Physics Chinese Academy of Sciences Shanghai 201204 China; ^3^ State Key Laboratory of Photovoltaic Science and Technology Fudan University Shanghai 200433 China; ^4^ Hangzhou Institute of Technology Xidian University Hangzhou 311200 China; ^5^ Department of Physics The Chinese University of Hong Kong Shatin New Territories Hong Kong 999077 China; ^6^ Key Laboratory of Advanced Display and System Applications of Ministry of Education Shanghai University Shanghai 200072 China

**Keywords:** GIWAXS, in situ, perovskite optoelectronic, solution spin‐coating

## Abstract

Solution‐processable perovskite‐based devices are potentially very interesting because of their relatively cheap fabrication cost but outstanding optoelectronic performance. However, the solution spin‐coating process involves complicated processes, including perovskite solution droplets, nucleation of perovskite, and formation of intermediate perovskite films, resulting in complicated crystallization pathways for perovskite films under annealing. Understanding and therefore controlling the fabrication process of perovskites is difficult. Recently, synchrotron radiation‐based in situ grazing‐incidence wide‐angle X‐ray scattering (GIWAXS) techniques, which possess the advantages of high collimation, high resolution, and high brightness, have enabled to bridge complicated perovskite structure information with device performance by revealing the real‐time crystallization pathways of perovskites during the spin‐coating process. Herein, the developments of synchrotron radiation‐based in situ GIWAXS are discussed in the study of the crystallization process of perovskites, especially revealing the important crystallization mechanisms of state‐of‐the‐art perovskite optoelectronic devices with high performance. At the end, several potential applications and challenges associated with in situ GIWAXS techniques for perovskite‐based devices are highlighted.

## Graphical abstract

Synchrotron radiation in situ GIWAXS combined with other high‐throughput characterization techniques can reveal perovskite crystallization at different transport layer interface scans in a rapid and real‐time manner. The performance parameters of perovskite films are quickly detected with changes in film optimization conditions, and the mechanism of their influence on device performance can be clarified.

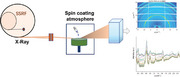

## 1 Introduction

Metal‐organic/inorganic halide perovskite materials have shown great application prospects in modern thin‐film optoelectronic devices such as solar cells,^[^ ^1^, ^2^, ^3^, ^4^ ^]^ X‐ray detectors,^[^ ^5^ ^]^ spintronic devices,^[^ ^6^ ^]^ and transistors^[^ ^7^ ^]^ owing to their widely tunable optoelectronic properties and simple solution processability. In recent years, solution spin coating has been an important and widely used method for the preparation of organic and perovskite‐based optoelectronic devices.^[^ ^1^, ^2^, ^3^, ^4^, ^5^, ^6^, ^7^ ^]^ The preparation of semiconductor thin films by solution spin coating is the foundation of the modern microelectronics industry. For instance, photoresistes for micromachining integrated circuits are deposited by spin‐coating because of the simplicity and low cost of the process.^[^ ^8^, ^9^, ^10^, ^11^ ^]^ On the road to improving the efficiency of PSCs, a crucial aspect is the optimization of thin‐film fabrication techniques, among which the one‐step antisolvent method is a commonly used deposition process for perovskite films.^[^ ^12^, ^13^ ^]^ The crystallization kinetics of perovskites during the one‐step spin‐coating process largely affect the final film quality in terms of phase purity, crystallinity, and film morphology and hence device performance.^[^ ^14^, ^15^, ^16^, ^17^, ^18^ ^]^ In comparison to the one‐step spin‐coating process, two‐step spin‐coating is another effective method for fabricating high‐performance PSCs, during which the perovskite crystallization process can be precisely controlled step by step. In addition to the spin‐coating process, the solution spin‐coating technique usually involves droplets, wet films, and a series of postprocessing conditions, giving rise to many complex crystallization pathways and making the fabrication process of optoelectronic thin films highly complicated and difficult to control.^[^ ^11^, ^12^, ^13^, ^14^, ^15^, ^16^ ^]^ The crystallization and orientation (in‐plane or out‐of‐plane) of spin‐coated semiconductor films often depend on the underlying substrate, and the films usually exhibit amorphous or polycrystalline states.^[^ ^17^, ^18^, ^19^, ^20^, ^21^, ^22^, ^23^, ^24^ ^]^ Therefore, obtaining high‐quality crystalline perovskite optoelectronic films and ultimately high‐efficiency and stable perovskite devices are highly challenging because of the complexity of the perovskite composition, dimensionality, and processing conditions.

A breakthrough in solving this problem lies in a comprehensive understanding of the intrinsic characteristics of perovskite materials and the intrinsic mechanism of their device operation, which is beneficial for improving the structure of perovskite photoelectric materials and the stability of devices by various means while also presenting a large challenge to traditional experimental means. Encouragingly, synchrotron radiation X‐ray technology, which has high collimation, high resolution, high brightness, and other characteristics, has unique advantages in multidimensional, cross‐scale, and high‐precision studies of material morphology and microstructure. It has become a powerful tool for materials research and a golden key to solving the response characteristics and stability problems of various new types of micro/nanodevices.^[^ ^12^, ^13^, ^14^, ^15^, ^16^, ^17^, ^18^, ^19^, ^20^, ^21^, ^22^, ^23^ ^]^

The state‐of‐the‐art Synchrotron radiation‐based in situ grazing‐incidence wide‐angle X‐ray scattering (GIWAXS) technique can enable one to bridge complex structural information with device output performance by revealing crystallization pathways during perovskite solution spin‐coating fabrication.^[^ ^13^, ^14^ ^]^ Kwan Wee Tan et al. showed a schematic representation of CsFA and MAFA formation during solution spin coating for different ratios of PVSK:DMSO and rH phases, involving 2, 4, and 6H perovskite phases.^[^ ^24^ ^]^ The intersections between the arrows and phase structures represent the possible structural phases that formed during the spin‐coating step to perovskite crystal formation. Moreover, we have proposed a series of synchrotron radiation experimental methods to study the relationship between the crystal structure of perovskite photoelectric materials and the photoelectric properties of devices.^[^ ^9^, ^10^, ^11^, ^12^, ^13^, ^14^, ^17^, ^18^, ^19^, ^20^, ^21^, ^22^, ^23^, ^24^, ^25^, ^26^, ^27^, ^28^ ^]^ For instance, the key mechanisms of surface crystalline phase purity, multi ordered crystallization, and interface defect passivation in highly stable optoelectronic materials and devices are revealed experimentally by Synchrotron radiation‐based GIWAXS; we have also developed a series of special observation platforms based on synchrotron radiation diffraction and scattering to observe the structure‐activity relationship of perovskite and other photoelectric thin films in real‐time to address the long‐standing problems of scientists in this area of research and support research teams to obtain many high‐level results.^[^ ^6^, ^28^, ^29^, ^30^, ^31^, ^32^, ^33^, ^34^, ^35^, ^36^, ^37^, ^38^, ^39^, ^40^, ^41^, ^42^, ^43^, ^44^, ^45^, ^46^, ^47^, ^48^, ^49^ ^]^

Focusing on the solution spin‐coating process, we herein illustrate how to obtain, perform and understand Synchrotron radiation‐based in situ GIWAXS for perovskite spin‐coating fabrication and summarize and assess recent results from Synchrotron radiation‐based in situ GIWAXS studies on versatile perovskite optoelectronic device systems in the spin‐coating process, aiming to elucidate the distinct features and common groundwork involved in film formation mechanisms and shedding light on future opportunities for employing Synchrotron radiation‐based in situ GIWAXS to study the fundamental working mechanisms of highly efficient and stable optoelectronic devices for mass production.

## 2 Discussion

### 2.1 Representative In Situ GIWAXS Setup for Perovskite Solution Spin‐Coating

Based on the synchrotron radiation facility, the combination of in situ GIWAXS technology and spin coating technology can reveal in depth the perovskite film formation and interface mechanism. Recently, a few synchrotron radiation facilities and teams have been able to carry out this kind of experiment, including the Shanghai Synchrotron Radiation Facility (SSRF),^[^ ^14^, ^15^, ^16^, ^17^, ^18^, ^19^, ^20^ ^]^ Advanced Photon Source (APS),^[^ ^8^ ^]^ Cornell High Energy Synchrotron Source (CHESS),^[^ ^9^, ^10^, ^11^ ^]^ and National Synchrotron Radiation Research Center (NSRRC).^[^ ^12^, ^13^ ^]^ Table S1 (Supporting Information) in support information lists the main conditions of in situ GIWAXS for the solution spin‐coating experiment setup at these four synchrotron radiation facilities. Based on the APS, the experimental setup of in situ GIWAXS with spin coating and a chamber, which contains a helium environment, a solution tube, and optical reflection, has been reported^[^ ^8^ ^]^ (Figure S15, Supporting Information). Lin X. Chen et al. improved the experimental setup and studied the mechanism of phase distribution in 2D halide perovskite films in a N_2_ atmosphere.^[^ ^9^ ^]^ The main experimental conditions are shown in the second line in Table S1 (Supporting Information), in which the key parameter is one‐step spin‐coating. However, the efficiency of perovskite solar cells was not reported in their work. Line 2 of Table S1 (Supporting Information) shows the experimental layout parameters of spin‐coating research using in situ GIWAXS technology based on the CHESS D1 beamline. Aram Amassian, Joshua J. Choi, and coworkers observed perovskite phase transitions by using an offline glovebox preparation solution,^[^ ^11^ ^]^ an online spin‐coating experiment, and kinetic stabilization.^[^ ^10^ ^]^ The third line in Table S1 (Supporting Information) gives the relevant experimental conditions of Aram Amassian's experiments. Line 3 of Table S1 (Supporting Information) demonstrates the in situ GIWAXS spin‐coating experiment based on the NSRRC, which included the experimental layout with the chamber in a N_2_ atmosphere without any control of ambient moisture or oxygen. Qin et al. reported the manipulation of mixed perovskite crystallization^[^ ^12^, ^13^ ^]^ and sequential A‐site doping of C_S_^+^ and Ga^+^, increasing the device efficiency to 23.5%.^[^ ^13^ ^]^ However, most of the above synchrotron radiation in situ GIWAXS studies were carried out in nitrogen or air, which is significantly different from the preparation conditions of perovskite solar cell devices in a standard glove box environment (such as the control of ambient water oxygen content), and it is difficult to fully and reliably determine the relationship between the crystallization process of the film and device performance.

Therefore, the establishment of synchrotron radiation in situ GIWAXS film‐forming observation equipment in a standard glove box environment will help solve the problems associated with in situ real‐time characterization of photoelectric films and device microstructures such as perovskites, which will provide reliable multidimensional and multilevel information for the system to determine the interface regulation behavior of thin films, the crystallization mechanism of perovskite materials and the structure‐activity relationships of devices. Based on the SSRF data, as shown in line 1 of Table S1 (Supporting Information), we established synchrotron radiation in situ GIWAXS observations of the film‐forming crystallization of semiconductor solutions based on the standard glove box environment, which can implement the diffraction and scattering signals of the phase change process of perovskite materials at various stages of perovskite precursor processing, including reaction, deposition, antisolvent spin coating, film formation, drying and annealing. The current synchrotron radiation in situ GIWAXS combined with other high‐throughput characterization techniques can also reveal perovskite crystallization at different transport layer interface scans in a rapid and real‐time manner. Moreover, the performance parameters of perovskite films are quickly detected with changes in film optimization conditions, etc., the preparation process of high‐quality perovskite films is revealed, and the mechanism of its influence on device performance is clarified.

**Figure 1:**
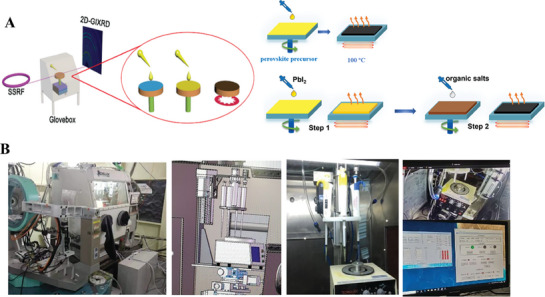
A) The in situ GIWAXS setups for solution spin‐coating at synchrotron radiation facilities. B) The setup @SSRF, with preparation conditions in a standard glovebox filled with N_2_ to avoid influences from ambient moisture and oxygen (*c* (H_2_O, O_2_) < 1 ppm). Reproduced with permission. Copyright 2021, The Royal Society of Chemistry.^[^ ^14^ ^]^

**Figure** **1** shows the basic layout and experimental flow of the line station layout and the in situ glove box device; that is, the synchrotron radiation quasi‐monochromatic X‐ray beamline is introduced into the experimental shed through the optical shack, the homogenizer platform is illuminated to the inside of the glove box through the beryllium window on the left side of the glove box, and the diffraction or scattering signal is collected by a 2D detector (CCD or Pilatus). Figure 1A shows the experimental layout of spin‐coating research using in situ GIWAXS technology based on the SSRF. The one‐step spin‐coating process and the two‐step spin‐coating process can be implemented separately. At the beamline stations of the SSRF, an in situ GIWAXS experimental setup, as shown in Figure 1B, is installed by integrating an N_2_‐filled glovebox containing a spin‐coater in the beamline.

Figure 1B shows photos of the in situ GIWAXS experiment setup, which contains several core components, such as an automatic drip system and a remote control system. As shown in Figure 1B, which shows the inside of the glove box, the homogenizer was placed on the workbench, the sample table was moved left and right, front and back, and up and down; at the same time, the homogenizer swung left and right and back and forth, cut the light, leveled the sample and achieved a certain grazing incidence angle incident on the sample surface. The top of the glove box was also equipped with a monitoring system, and the solution inside the glove box was observed through an external display. To an obvious monitor, there is a lighting system above the box and a drip pump interface. An optical glass window at the bottom of the glove box was set up for light experiments; the back of the glove box was equipped with sensors, purification interfaces, and access ports; the right side of the glove box was equipped with a Capeton film window to ensure the acquisition of X‐ray signals at large angles and that the Capeton film was < 0.25 mm thick, which can reduce the loss of X‐ray transmission signals.

Figure 1B also shows the automatic spin coating injection system, which is remotely controlled by a computer fully automatic cylinder and turntable through four microprecision pipette guns to achieve sequential automatic drip, which not only saves a large number of precursor solutions but can also precisely control the solution drop size and drop dosing time and can achieve continuous drop addition of four solutions; the homogenizer is remotely controlled by the computer to realize the spin coating process synchronously. These setups provide the possibility for studying the complex interface in the multistep film formation process, nucleation process, and crystallization process. By virtue of this standard glovebox, both the environmental atmosphere (e.g., temperature, humidity, oxygen, light, etc.) and film deposition process (e.g., spinning speed, antisolvent, thermal annealing, etc.) can be precisely controlled and modulated.^[^ ^14^, ^15^, ^16^, ^17^, ^18^, ^19^, ^20^ ^]^

### 2.2 3D Perovskite Process Regulation During Spin‐Coating Monitored by In Situ GIWAXS

The growth interface of perovskites on electron and hole transport layers (ETLs and HTLs) plays a direct and key role in the formation of perovskites during the solution spin coating process, including a series of film‐forming problems such as interfacial nucleation and orientation growth. We have made remarkable progress in terms of modified ETLs, which have shown that improving the SnO_2_ bottom interlayer could significantly enhance perovskite crystallization and buried interface contact and subsequently increase the performance of the resulting PSCs.^[^ ^10^, ^11^, ^12^, ^13^, ^14^ ^]^

**Figure 2:**
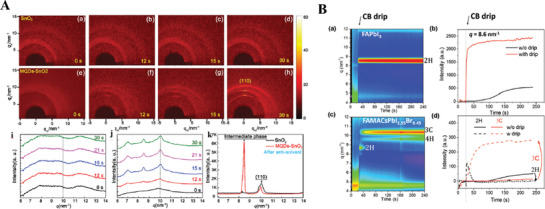
A) Time evolution of in situ GIWAXS patterns of deposited perovskite films during the spin‐coating process (a–g); integrated in situ XRD spectra of perovskite films deposited on ETLs of SnO_2_ (i) and MQDs‐SnO_2_ (j) before antisolvent treatment; (k) perovskite films integrated with 1D XRD spectra after antisolvent treatment. Reproduced with permission. Copyright 2021, The Royal Society of Chemistry.^[^ ^14^ ^]^ B) In situ GIWAXS maps of (a) FAPbI_3_ and (c) FAMACsPbI_2.55_Br_0.45_ precursors with a CB antisolvent drip of ≈20 s for spin‐coating times up to 240 s. Time evolution of the diffraction patterns corresponding to the 2H phase (q ≈8.6 nm^−1^) and perovskite 3C phase (q ≈10.1 nm^−1^) for (b) FAPbI_3_ and (d) FAMACsPbI_2.55_Br_0.45_. Reproduced with permission. Copyright 2019, Wiley‐VCH.^[^ ^10^ ^]^

**Figure** **2**A shows the in situ GIWAXS patterns of perovskite films deposited on pristine SnO_2_ (a–d) and MQD‐SnO_2_ (e–h) ETLs during the spin‐coating process.^[^ ^14^ ^]^ In comparison to the GIWAXS patterns of perovskite films deposited on pristine SnO_2_ ETLs (c,d), the perovskite films deposited on MQD‐SnO_2_ ETLs had bright diffraction rings and spots in (g,h), respectively. Before the antisolvent treatment, the perovskite film deposited on the MQD‐SnO_2_ ETL underwent rapid nucleation. Movies S1 and S2 (Supporting Information in ref. [14]) display the whole evolution process of in situ GIWAXS patterns as a function of spin‐coating time.

The GIWAXS patterns of the perovskites deposited on the pristine SnO_2_ ETL and MQD‐SnO_2_ ETL are also shown azimuthally as the integrated in situ 1D GIWAXS spectra in Figure 2A(i,j), respectively. Figure 2A(i)shows no obvious peaks but rather several broad peaks, suggesting that there are no perovskite crystals before the antisolvent drips into the perovskite precursor. In Figure 2A(j), the peaks at q≈7.0 nm^−1^ and q≈8.5 nm^−1^ are assigned to perovskite intermediate phases, and the peak at q≈10.0 nm^−1^ is ascribed to the (110) plane of the tetragonal perovskite phase. These three peaks became increasingly notable during the 12–15 s time period and then remained almost constant until 30 s, providing an appropriate window for subsequent antisolvent treatment. Furthermore, the integrated 1D GIXRD spectra of the reference and target perovskite films after antisolvent treatment are shown in Figure 2A(k). The lower peak at q = 10.0 nm^−1^ for both films indicates a low number of perovskite crystals. However, for perovskite films deposited on the MQD‐SnO_2_ ETL, the peak at q≈8.5 nm^−1^ is higher than that on the SnO_2_ ETL, which indicates that the intermediate perovskite phase dominates the whole spin‐coating period after antisolvent treatment. Therefore, the experimental results obtained for perovskite precursor‐doped quantum dot materials, including graphene oxides and Ge_2_O, support that an intermediate phase is crucial for the final crystallization of perovskite.^[^ ^10^, ^11^ ^]^

For the FAPbI_3_ perovskite precursor during spin coating, antisolvent spin coating is an extremely effective method for rapid film‐forming crystallization of the perovskite precursor. However, due to the short duration of spin coating with an antisolvent, this reaction process is difficult to monitor in real‐time, and the corresponding reaction mechanism is also difficult to clearly reveal. Figure 2B(a) shows the time‐resolved in situ GIWXAS patterns during the spin‐coating process. The peak corresponding to the 2H phase (q ≈0.83 Å^−1^) appeared and rose rapidly upon antisolvent dripping at the 20th s and then maintained a high peak intensity throughout the whole 240 s process. Moreover, the different intensities of the time‐dependent relationships between the antisolvent treatment and not can be seen in Figure 2B(b). These results show that the rapidly increasing intensity indicates that the FAPbI_3_ 2H phase was formed immediately after the antisolvent treatment was applied at 20 s. Compared with the mechanism of crystallization kinetics of the 2H phase without CB dripping, the dramatic effect of the drip instantaneously stimulates the nucleation and growth of this crystalline phase, and the CB drip promotes nucleation and growth by strongly suppressing the complex solvent during the sol–gel film stage.^[^ ^10^ ^]^

The in situ GWXAS results for the case of the FAMACsPbI_2.55_Br_0.45_ precursor solution are shown in Figure 2B(c). First, CB addition also promoted 2H phase formation, but the 2H phase slowly disappeared as the 3H and 4H phases gradually appeared during the 20 s. Furthermore, the 3H and 4H phases became increasingly intense as time progressed. Figure 2B(d) shows the dynamics of the 2H phase and 3C evolution without and with CB antisolvent treatment during the time from the 40th to the 240th s.^[^ ^10^ ^]^

**Figure 3:**
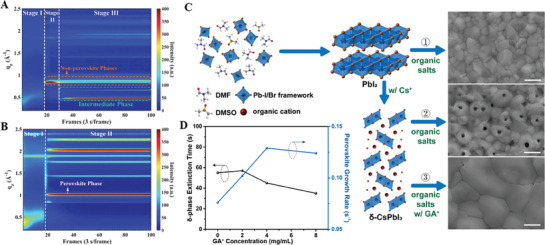
Crystallization kinetics of perovskites A) without and B) with Cs^+^ doping during the one‐step spin‐coating process unveiled by in situ GIWAXS. C) Three‐phase transition pathways for perovskite films during the two‐step spin‐coating process and the corresponding SEM images. D) δ‐CsPbI_3_ phase extinction time and perovskite growth rate for different concentrations of GA^+^. Reproduced with permission. Copyright 2019, 2020, 2021, Wiley‐VCH.^[^ ^12^, ^13^ ^]^

The addition of anionic Lewis acid or alkali ions to the precursor of perovskite can effectively improve the nucleation and crystallization of perovskite.^[^ ^12^, ^13^, ^14^, ^15^, ^16^, ^17^ ^]^ The solvents and solutes involved in this process are complex; therefore, real‐time observation via in situ GIWAXS is crucial for studying the regulation and synthesis mechanism of thin films. With the aid of in situ GIWAXS characterization, we systematically investigated the detailed crystallization process of mixed perovskite MA_0.17_FA_0.83_Pb(I_0.83_Br_0.17_)_3_ without and with Cs^+^ doping during one‐step spin‐coating.^[^ ^12^ ^]^ Three crystallization stages were identified, namely, precursor solution (stage I), hexagonal δ‐phase (2H) (stage II), complex phases comprising hexagonal polytypes (4H, 6H), MAI‐PbI_2_‐DMSO intermediate phases, and perovskite α‐phase (stage III), as shown in **Figure** **3**A. Combined with the corresponding device performance and ex situ characterizations, they further proposed the concept of an “annealing window” covering the duration of stage II: the as‐cast film should be annealed within the annealing window to bypass the formation of hexagonal polytypes (stage III), thus achieving decent device performance. Remarkably, the crystallization pathway can be manipulated by incorporating Cs^+^ in mixed perovskites (Figure 3B), which can circumvent the formation of secondary phases in stage III by promoting the formation of the α‐phase both kinetically and thermodynamically, thereby significantly extending the annealing window.

In comparison to the one‐step spin‐coating process, two‐step spin‐coating is another effective method for fabricating high‐performance PSCs (Figure 1A),^[^ ^13^ ^]^ during which the perovskite crystallization process can be precisely controlled step by step through sequential doping of Cs^+^ and GA^+^, as shown in Figure 3C.^[^ ^13^ ^]^ The incorporation of Cs^+^ in the first step leads to the formation of the δ‐CsPbI_3_ phase, which serves as a nucleation center in the second step. Nevertheless, sparse δ‐CsPbI_3_ crystals cause nonuniform nucleation and hence pinholes in the film. Fortunately, GA^+^ doping in the second step not only accelerates the phase transition from δ‐CsPbI_3_ to perovskite but also greatly eliminates pinholes and improves the crystal grain size, thus increasing the efficiency of PSCs to 23.5%.^[^ ^13^ ^]^

### 2.3 2D Perovskite Nucleation Regulation During Spin‐Coating Monitored by In Situ GIWAXS

Owing to the outstanding nucleation properties of 2D perovskites and the easily regulated crystallization orientation, a large number of research teams from various countries have made great efforts in recent years, including component regulation and growth interface optimization of 2D perovskite precursors.^[^ ^15^, ^16^, ^17^, ^26^, ^27^, ^28^, ^29^, ^30^, ^31^, ^32^ ^]^ In recent years, the reduced‐dimensional metal halide perovskites (RDPs) have attracted significant attention due to their promising light harvesting, emissive properties, and environmental stabilities, Sargent's group has reported that layered intermediate complexes formed with the solvent provide a scaffold that facilitates the nucleation and growth of RDPs during annealing, as observed via in situ GIWAXS.^[^ ^26^ ^]^ Meanwhile, we have conducted systematic in situ GIWAXS characterization during spin coating on different types of samples and shown the evolution of the structure as a function of time and additives. The spin‐coating process was conducted under the same preparation conditions in a glovebox and was protected by N_2_ flow to avoid influences from ambient moisture and oxygen. The samples were probed by a beam light of 10 keV synchrotron radiation with incident angles of 0.1 and 0.4 degrees to ensure surface and bulk penetration, respectively.

**Figure 4:**
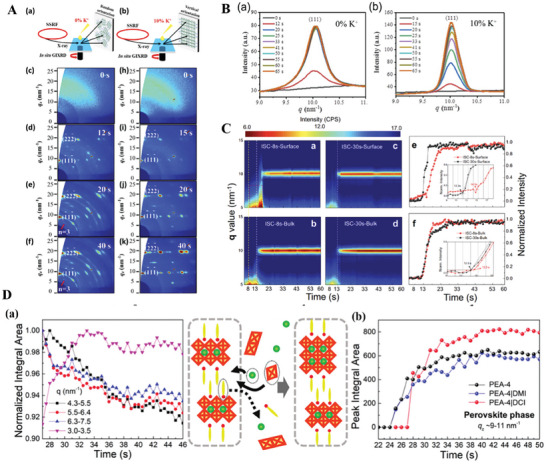
A): In situ GIWAXS patterns of quasi2D perovskite thin films without K^+^ in the spin coating process from 0 to 60 s. (a) Schematic diagram of Synchrotron radiation‐based in situ GIWAXS characterization for quasi2D perovskite thin films without K^+^. (b) Schematic diagram of in situ GIWAXS characterization of 10%‐doped quasi2D perovskite thin films. (c) 0 s, (d) 12 s, (e) 20 s, and (f) 40 s. Red arrows represent low *n*‐number intermediate phases. In situ GIXRD patterns of 10% K^+^‐doped quasi2D perovskite thin films obtained during the spin‐coating process from 0 to 60 s. (h) 0 s, (i) 15 s, (j) 20 s, and (k) 40 s. B): Perovskite diffraction peak (111) for the (a) 0% K^+^ film and (b) 10% K^+^ film for different spin‐coating elapsed times. C) In situ crystallization comparison of the surface and bulk areas between the optimal ISC‐8s and SC methods by in situ GIWAXS. D): (a) Phase buffering: Low‐*n* phase intensity evolution during the crystallization process in DCI‐treated PEA‐4 samples and the corresponding scheme. (b) Perovskite peak integral area of neat PEA‐4, DMI‐ and DCI‐treated PEA‐4 from *q*_z_ ≈9–11 nm^−1^. Reproduced with permission. Copyright 2020, American Chemical Society; Copyright 2021, The Royal Society of Chemistry.^[^ ^15^, ^16^, ^17^ ^]^

In **Figure** **4**A, we simply incorporated potassium ions (K^+^) into a quasi2D precursor solution, which can dramatically change the nucleation steps during the perovskite film spin‐coating process, as revealed by in situ GIWAXS.^[^ ^16^ ^]^ Notably, a desired vertically oriented 2D phase without an intermediate compound can be easily formed after spin coating, which simultaneously reduces the distribution of low *n*‐number 2D perovskite phases in association with suppressed trap states. As shown in Figure 4B, according to the perovskite diffraction peaks (111) for the 0% K^+^ and 10% K^+^ films with different spin‐coating times, the intensity of the diffraction peak (111) of the 0% K^+^ film reaches its saturation point at ≈20 s. However, the intensity of the 10% K^+^ film reaches a similar value at 20 s compared with that of the 0% K^+^ film and further increases until the end of the spin‐coating process. These results indicate the better crystallinity of the K+‐doped film.

According to our recent results,^[^ ^15^ ^]^ the in situ GIWAXS characterization properties of 2D PVSK growth on optimal ISC‐8‐ and SC‐treated samples are shown in Figure 4C. A false‐color map versus the *q* value and time is plotted in Figure 4C (a–d) with real data. In the first 8 s, both the ISC‐8 and 30 s samples exhibit broad scattering intensities of ≈5 nm^−1^, which originate from the precursor solution.^[^ ^10^ ^]^ As spin‐coating proceeds, the dispersed ring starts to expand as a result of rapid evaporation of the solvent (please refer to 0–8 s in Videos S1–S4, Supporting Information in the ref. [15]), implying the formation of intermediate phases. Moreover, no noticeable peak at ≈10 nm^−1^ is observed, indicating that no PVSK phase is generated at this stage. When spin coating ceased at 8 s, a discontinuous area immediately appeared for the surface areas of the ISC‐8 sample, as highlighted in the red dotted box in Figure 4C(e), which suggested that the sudden decrease in rotation retarded crystallization in the ISC‐8 sample. This metastable region does not transform into PVSK phases until 14.5 s, as determined from the line cuts at ≈10 nm^−1^ in Figure 4C(e). Since these areas still fall within the range of nonperovskite precursor phases, it is clear that the surface region of ISC‐8s does not crystallize before 14.5 s. On the other hand, the PVSK peak is identified in the bulky area at 12.3 s, as shown in Figure S1b,f (Supporting Information) in our previous report;^[^ ^15^ ^]^ this peak is ≈2 s earlier than the surface and directly reinforces our argument in the manuscript; that is, the ISC method facilitates interface initialized crystallization.^[^ ^15^ ^]^

For the SC samples, the PVSK signals were already generated at ≈12 s in both the bulk and surface regions of the SC samples. Since the signals from the bulk contain information from the surface, we are unable to distinguish which of these signals acts as the initial site for nucleation from in situ data. However, the *n*‐value and orientation trend in the TOF‐SIMS and ex situ‐GIWAXS results indicate that bidirectional crystallization is dominant at the interface. Notably, if we look into the surface area of the ISC‐8s and SC samples, the PVSK signal is located at 10 and 10.1 nm^−1^ for the ISC‐8s and SC samples, respectively, which indicates smaller *n* values in the former and agrees with the results of TOF‐SIMS.^[^ ^15^ ^]^

Furthermore, as shown in Figure 4D, we recently reported that highly ordered lattice arrangements in 2D lead halide perovskites, which are examples of paradigm PEA spacers, can be acquired by the addition of 4,5‐dicyanoimidazole (DCI) only after solution spin coating with no posttreatment process. A series of in situ GIWAXS and high‐resolution transmission electron microscopy (HR‐TEM) measurements have revealed the epitaxial growth of perovskite phases that gradually buffer the internal lattice strain and consequently regulate the lattice orientation, leading to a decrease in the trap density and prolongation of the carrier lifetime.^[^ ^17^ ^]^

### 2.4 Perovskite Quantum Dots Nucleation Regulating in Spin‐Coating Monitored by In Situ GIWAXS

Perovskite quantum dots (QDs) generally exhibit size‐dependent optoelectronic behaviors,^[^ ^48^ ^]^ enabling effectively tuned photon emission: CsPbBr_3_ QDs with size arranges of 3–5 nano‐meters are required owing to the exciton Bohr diameter of CsPbBr_3_ ≈7  nm.^[^ ^49^ ^]^ However, it has been reported that it is difficult to achieve the mono‐dispersed sub‐5  nm‐sized QDs using traditional colloidal synthesis methods. The ligands on the QDs surface are readily lost upon ligand exchange in the process of atoms' self‐assembly into semiconducting nanoparticle solids, which would be the result of the variable degrees of QD fusion and therefore increase their polydispersity. Two key steps‐ligand exchange and coupling during film formation are particularly the challenges in dealing with ultraconfined dots, leading to a clear redshift and meanwhile increasing the emission linewidth.^[^ ^48^ ^]^ Therefore it is pursued a process that would avoid this approach to materials processing, and find an effective method to fabricate the QDs film in one step using spin‐coating. However, synthesizing perovskite QD solids on a range of substrates, which are monodispersed and suitably coupled, is a notorious challenge: the ligand's structure influences the dimensionality of the finally assembled perovskites, inevitably producing variously 3D networks, 2D quantum wells, and 1D chains. These above problems motivated us to discuss in more detail that how the structure of ligands can regulate perovskite QD synthesis‐on‐substrate (SoS) to form the colloid films.^[^ ^48^ ^]^

**Figure 5:**
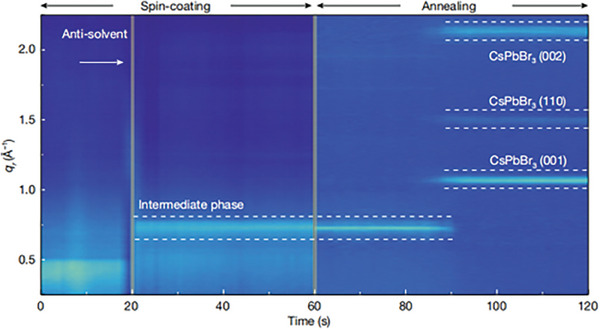
Formation of Synthesis‐on‐substrate of QD films revealed by In situ GIWAXS. Reproduced with permission. Copyright 2022, Springer Nature.^[^ ^48^ ^]^

With the aid of synchrotron irradiation in situ GIWAXS, the CsPbBr_3_ QD formations were studied via synthesis‐on‐substrate.^[^ ^48^ ^]^ As shown in **Figure** **5**, three distinct stages of film growth have been identified. The broad and weak scattering ring located at *q*_r_ ≈0.43 Å^−1^ appeared first, being assigned to the precursor phase of perovskite solution. After the anti‐solvent dripping, a new scattering peak located at *q*_r_ ≈0.76 Å^−1^ gradually emerged, which is assigned to the intermediate phase, marked by PbBr_2_–2∙DMSO. A transformation of the crystalline phase has been observed upon heating, with three distinct peaks indexed to the cubic CsPbBr_3_ phase progressively appearing and intensifying. We found that the meta‐stable perovskite intermediate phase via controlling the crystallization process contributed to the growth of pure phase CsPbBr_3_ QDs with narrower size distribution.^[^ ^48^ ^]^

### 2.5 Crystallization Mechanisms for Perovskite Spin‐Coating Fabrication

**Figure 6:**
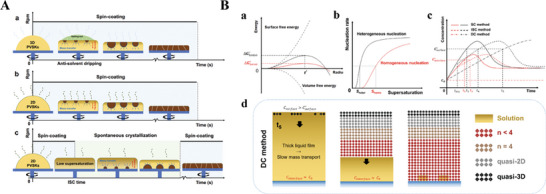
A) Illustration of top‐down and bottom‐up crystallization. Conventional downward crystallization by (a) antisolvent dripping in 3D PVSKs, (b) direct spin coating in 2D PVSKs, and (c) dominant upward crystallization by the ISC method in 2D PVSKs. Reproduced with permission.^[^ ^15^ ^]^ B) Comparison of film crystallization among three spin‐coating, ISC, and direct‐dripping methods. (a) Precursor concentration evolution and (b) mechanistic illustration of the crystallization processes. Reproduced with permission. Copyright 2021, Springer Nature.^[^ ^15^ ^]^

For 3D PVSKs prepared using solution spin‐coating fabrication, downward growth is widely acknowledged to prevail owing to the high supersaturation at the surface resulting from spin‐coating and the antisolvent salting‐out effect,^[^ ^26^, ^27^, ^28^, ^29^, ^30^, ^31^, ^32^ ^]^ as illustrated in **Figure 6**A(a). In regard to the 2D PVSKs grown by solution spin‐coating methods, the introduced larger organic cations will be incorporated into the crystal lattice instead of serving as an extra additive, which naturally changes the solvent evaporation rate, precursor unit bonding process, and hence the film nucleation kinetics.^[^ ^15^ ^]^ Several pioneering works have provided important observations on the crystallization of 2D PVSKs. For example, Zhou's group found that the initial formation of low‐*n* (*n* < 4) phases in BA_2_MA_3_Pb_4_I_13_ is followed by high‐*n* phases during spin coating via in situ photoluminescence (PL) spectroscopy, indicative of the favorable kinetics of LD PVSKs despite their higher formation energy than 3D PVSKs.^[^ ^10^, ^15^ ^]^ In contrast, Kanatzidis and coworkers, with operando GIWAXS technology, recently confirmed that BA_2_MA_2_Pb_3_I_10_ features downward crystallization consisting of the first highly oriented 3D‐like phases at the surface and subsequent growth of less oriented LD phases in the bulk (Figure 6A(b)). The divergence between these methods may be due to either stoichiometric differences or preparation method variations, which further underlines the complexity of perovskite crystallization kinetics even in the same organic cation‐based 2D systems. Therefore, a universal interpretation of what dictates nucleation and crystal growth in 2D PVSKs is still lacking. In our recent reports, we accidentally disclosed the crystallization process in layered 2D PVSKs by Synchrotron radiation‐based in situ GIWAXS during the spin‐coating process. For instance, the PEA_2_MA_3_Pb_4_I_13_ paradigm can be significantly modulated by the intermittent spin‐coating (ISC) method, as shown in Figure 6A(c). The precursor solution is first spin‐coated for a certain period, namely, the ISC time. Then, the spin process ceases, and the transparent wet film is allowed to stand until its color changes, which is immediately finalized by another spin coating. These three‐stage film‐forming processes play different roles. The first procedure homogenizes the precursor solution and sets the initial supersaturation difference between the surface and the interface upon entering the second stage, during which the precursor mass transfer driven by the concentration gradient and spontaneous solvent evaporation modulate the supersaturation rate both on the surface and at the interface, leading to interface‐initialized nucleation and dominant upward crystal growth. The last spin process ensures the fast removal of residual solvent, which prevents the redissolution of as‐formed crystals and hence prevents the impairment of film morphology.

Based on all the results in our previous report,^[^ ^15^ ^]^ we obtained a complete picture of crystallization during spin‐coating, ISC, and direct‐dripping methods. As schematically shown in Figure 6B, during the spin‐coating process, the concentration at the surface (C_s_) rapidly increases with the intense evaporation of perovskite solvents, while the concentration at the interface (C_i_) increases more slowly through mass transfer from the surface driven by the concentration difference. The high supersaturation at the surface and the low nucleation barrier at the interface result in nearly simultaneous nucleation at both sides (t_1_ = t_2_ in Figure 6B). Given the much more intense solvent evaporation during spin‐coating, the subsequent growth follows the continuous crystallization model with a gradually decreasing *n* value and an attenuated orientation toward the center of the films. In the case of the ISC method, although C_s_ increases dramatically in the first stage of spin‐coating, the concentration growth rate dramatically decreases when spin‐coating ceases. At this stage, C_i_ will reach the critical supersaturation level earlier than C_s_ (t_3_ < t_4_), and the overall crystallization is thermodynamically controlled since the film remains at low evaporation rates. Therefore, the crystal alignment will be much more oriented than that of spin‐coating. Moreover, the prolonged crystallization in the ISC method ensures sufficient time for mass transfer from the surface to the interface (i.e., t_3_ > t_2_), which effectively eliminates the supersaturation differences and suppresses nucleation at the surface. Although interfacial nucleation occurs first in both the spin‐coating and ISC methods, the initial nucleation time at the surface is much longer than that at the interface, indicating that upward crystallization consumes most of the MA cations. Therefore, the stoichiometric *n* value in the precursor solution decreases, and the subsequent surface crystallization yields lower *n* phases than those on the surface. As the ISC time decreases to 0 s (that is, direct dripping), the crystallization may be completely different. The surface area undergoes spontaneous evaporation similar to that in the second stage of the ISC method. However, the concentration gradient between the surface and interface is significantly reduced because of the thick liquid film, and thus, the mass transfer is limited. As a result, C_i_ remains nearly invariant, while C_s_ gradually increases to the critical supersaturation for homogenous nucleation (t_5_), leading to the well‐known downward crystallization. Moreover, the morphology becomes more mature and rough as the ISC time becomes shorter than 8 s, suggesting that unidirectional downward crystallization is not conducive to the formation of smooth films.

### 2.6 In Situ GIWAXS Monitoring of Perovskite Post treatment After Spin‐Coating

**Figure 7:**
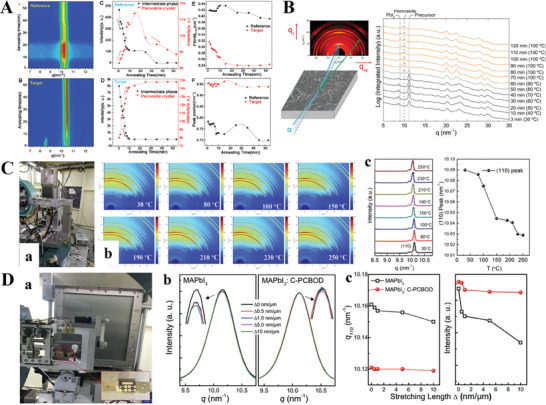
A) In situ GIWAXS measurements of perovskite films during the annealing process. The integrated 1D‐GIXRD spectra of reference (a) and target (b) perovskite films at different annealing times; diffraction intensity (peak area) of the reference (c) and target (d) perovskite films at different annealing times; FWHM (e) and peak position (f) of the perovskite (110) diffraction peak of both the reference and target perovskite films plotted as functions of annealing time. B) In situ GIWAXS was used to investigate the structural evolution and film morphology of methylammonium lead triiodide/chloride (CH_3_NH_3_PbI_3‐x_Cl_x_) in mesoporous block copolymer‐derived alumina superstructures during thermal annealing. C) In situ GIWAXS measurements of the perovskite films during thermal treatment. (a) A photograph of the experimental setup for the temperature‐dependent Synchrotron radiation‐based in situ GIWAXS measurements. (b) 2D GIWAXS patterns of beta‐CsPbI_3_ perovskite films annealed at different temperatures (30, 80, 100, 150, 190, 210, 230, and 250 °C). D) In situ GIWAXS measurements of the mechanical durability of flexible PSCs. (a) A photograph of the setup for the in situ stretching Synchrotron radiation‐based GIXRD experiments, where the inset shows a flexible PSC to be mounted for the measurements; (b) The intensity and (c) position of the diffraction peaks of the MAPbI_3_ and MAPbI_3_:C‐PCBOD films under various stretching lengths. Reproduced with permission. Copyright 2021, The Royal Society of Chemistry; Copyright 2019, Science AAAS; Copyright 2019, Wiley‐VCH; Copyright 2014, American Chemical Society.^[^ ^4^, ^14^, ^21^, ^24^ ^]^

Post‐treatment of perovskite films after spin coating is also critical for further improving device performance. Various research groups have recently developed a series of new and effective post‐treatment processes, such as traditional annealing, solvent atmosphere annealing, vacuum flash annealing, an appropriate humidity atmosphere, and oil immersion annealing.^[^ ^30^, ^31^, ^32^, ^33^, ^34^, ^35^, ^36^, ^37^, ^38^, ^39^, ^40^ ^]^ As shown in **Figure** **7A**, we performed in situ real‐time GIWAXS measurements during the subsequent annealing process to investigate the transformation from the intermediate phase to the final perovskite phase.^[^ ^14^ ^]^ The annealing process consisted of a gradual increase in the annealing temperature from room temperature to 100 °C within 2 min (stage I), a temperature plateau at 100 °C for 10 min (stage II), and another temperature increase to 150 °C for 35 min (stage III). Figure 7A(A,B) shows the azimuthally integrated intensity profiles derived from the GIWAXS patterns, demonstrating a gradual structural evolution from distinct intermediate perovskite crystals into highly ordered perovskite crystals during the annealing process. As shown in Figure 7B(a), Tan et al.^[^ ^24^ ^]^ reported that the temperature during the heating stage was increased by 10 °C every 10 min during the annealing of perovskite films in N_2_. It took ≈2–3 min for the heated stage to reach the set temperature. The integrated intensity plots displayed in Figure 7B(b) reveal the TTD structural evolution of the MBCP‐Al_2_O_3_ perovskite sample during a 120 min annealing process as the temperature increased from 30 to 100 °C. At the beginning of the in situ GIWAXS experiment, the crystallinity of the precursor was detected in the 2D profile at the peak at q ≈11 nm^−1^. This structural transition was completed at the 60 min time point, similar to the 10 min time point for the ITD‐annealed MBCP‐Al_2_O_3_ perovskite at 100 °C in nitrogen. Perovskite film degradation was observed at the 70th min when the temperature was increased to 100 °C. Like in the ITD annealing process under nitrogen, three distinct structures were identified during the TTD process of CH_3_NH_3_PbI_3‐x_Cl_x_ in the MBCP‐Al_2_O_3_ superstructures: 1) a crystalline precursor, 2) a perovskite, and 3) PbI_2_ as a perovskite degradation product.

In Figure 7C, we further developed a thermal treatment setup for in situ GIWAXS, which can work under specific conditions during standard tests of PSCs, such as RH (0–100%), atmosphere (N_2_, He, Ar), light exposure, and IV measurements. In a previous report in Science,^[^ ^4^ ^]^ we arranged temperature‐dependent in situ GIWAXS measurements for the beta‐CsPbI_3_ perovskite to reveal its thermal stability and mechanism. As shown in Figure 7C, we measured the GIWAXS at 30–250 °C and the temperature‐dependent q (q = 4 πsinθ λ^−1^) values, revealing that the *q* value of our black β‐CsPbI_3_ perovskite only exhibited ≈0.06 variation between 250 and 30 degrees. This small variation confirmed that the XRD patterns measured at different temperatures are very similar and will result in the transformation of the phase to yellow α‐CsPbI_3_. These results also demonstrated that our β‐CsPbI_3_ films are thermally stable, leading to the high efficiency and stability of inorganic PSCs.

In addition, we carried out a series of stretching experiments for PSCs with the aid of in situ Synchrotron radiation‐based GIWAXS (Figure 7D(a)) to reveal the underlying reasons for the device performance decrease induced by the applied tensile force‐inflexible PSCs. Moreover, the structural differences between these two PSCs after treatment with different precursor additives or antisolvent^[^ ^18^, ^21^ ^]^ were investigated. Figure 7D(b) shows the perovskite (110) diffraction peak profiles measured for these two PSCs under different tensile forces. Figure 7D(c) shows that the perovskite (110) diffraction peak intensity and position change as a function of the stretching distance. There was almost no significant change in peak position or intensity after doping with C‐PCBOD, indicating that the structural stability of the doped polycrystalline CH3NH3PbI3 film increased with increasing external load stress. However, the undoped MAPbI_3_ film has a significant change in the peak position and intensity of the perovskite (110) diffraction peak.^[^ ^21^ ^]^ This indicates that collision and wear occur between the perovskite grains during the stretching and bending processes, resulting in a change in the crystal structure characteristics of the perovskite polycrystalline film. With the soft C‐PCBOD filling at the grain boundaries, the collision and wear between the perovskite grains are reduced, and thereby, their crystal characteristics are hardly changed.^[^ ^21^ ^]^ In brief, the doped C‐PCBOD molecule can minimize film defects by filling boundaries and hinging perovskite grains, thus enhancing the mechanical stability of flexible MAPbI3:C‐PCBOD‐based perovskite solar cells. This method provides a practical solution for enhancing the intrinsic PSC mechanical stability and promotes the large‐scale fabrication of flexible perovskite‐based devices.

To better understand the interface, that is perovskite precursor deposition surface, we have developed a novel characterization technique based on Synchrotron radiation‐based GIWAXS, which is successfully applied in flexible PSCs.^[^ ^43^ ^]^ We find that the Synchrotron radiation‐based X‐rays are capable of penetrating the PEN substrate, allowing them to access the ETL and perovskite layers from the PEN side. The measurement results reveal that perovskite deposited on the optimized ETL exhibits no significant precursor phase peak at q≈9.3 nm^−1^ when measured at a smaller incidence angle of 0.2°, in contrast to an incidence angle of 0.4°. This indicates a higher crystal phase purity at the interface between the perovskite layer and optimized ETL, resulting in a PCE of 23.57% for the FPSC with CQD‐SnO2 serving as the ETL. Notably, this method proposed the first instance of acquiring X‐ray diffraction patterns without any damage from buried perovskite/ETL interfaces using GIWAXS.^[^ ^43^ ^]^

## 3 Summary and Outlook

In summary, Synchrotron radiation‐based in situ GIWAXS for metal halide perovskite spin‐coating fabrication has undergone significant development, which promotes the understanding of perovskite nucleation and crystallization mechanisms and provides guidance for improving the fabrication technology for high‐performance PSCs. We found that perovskite intermediate phases corresponding to the yellow phases and PbX2 are first formed during the 3D perovskite spin‐coating fabrication, otherwise, the lower n‐value phases (<3) are easily formed in the fabrication of 1D and 2D perovskite during spin‐coating process.

Despite the impressive efficiency of PSCs, the toxicity of lead (Pb) in perovskites has been an obstacle hindering their commercialization.^[^ ^9^ ^]^ To address this issue, tin‐based perovskites, which possess a smaller bandgap, are receiving increasing attention in the photovoltaic field.^[^ ^10^ ^]^ However, the crystallization process of tin‐based perovskites is much faster and more uncontrollable than that of their Pb‐based counterparts due to the higher reactivity of the Lewis acidity of SnI_2_, resulting in poor film quality and thus inferior device performance. Therefore, an in‐depth understanding of the crystallization mechanism by virtue of in situ GIWAXS techniques is imperative for guiding the optimization of the film‐formation process of tin‐based perovskites. Nonetheless, the oxidation of Sn^2+^ in perovskites to Sn^4+^ challenges the implementation of in situ GIWAXS experiments; therefore, a vacuum or N_2_‐filled airtight chamber is a prerequisite for mimicking the real film deposition process in a glovebox. Moreover, the spin‐coating process should be extremely steady during in situ GIWAXS measurements to acquire high‐quality data because of the grazing incidence geometry of the testing setup. In addition, the crystallization process of perovskites under various atmospheres is also worth studying to understand the influences of external stresses on perovskites and the degradation mechanism. Therefore, a setup that can conduct in situ GIWAXS measurements under different conditions (e.g., moisture, oxygen, heat, and light) is highly desirable.^[^ ^38^, ^39^, ^40^, ^41^, ^42^, ^43^, ^44^, ^45^ ^]^ These in situ GIWAXS techniques would also provide important guidance for the fabrication of highly luminescent perovskite films and high‐performance perovskite light‐emitting diodes (LEDs).^[^ ^6^, ^46^, ^47^, ^48^, ^49^ ^]^ At present, perovskite LEDs are striving to improve device stability, however, the real‐time degradation mechanism of perovskites, especially low‐dimensional perovskites with complex compositions, under the synergistic effect of an electric field and other conditions (e.g., moisture, oxygen, light, etc.) has not been fully characterized. In addition, the dimensionality of mixed‐dimensional perovskites can be tailored with the help of monitoring the crystallization behavior via in situ GIWAXS, which is highly important for realizing blue, red and NIR perovskite LEDs with fine control over the exciton energy.^[^ ^6^, ^46^, ^47^, ^48^, ^49^ ^]^

Synchrotron radiation‐based in situ GIWAXS combined with current typical spectrum measurements (such as TRPL, PL, transient absorption spectrum, and Raman spectrum) can be used to establish a real‐time synergistic effect between the microstructure and energy level structure of organic and perovskite films during the solution spin‐coating or slot‐die coating stages,^[^ ^50^, ^51^, ^52^ ^]^ and during humid and thermal (such as 85% RH, 85~150 °C) environment treatment to monitor the micro‐structural evolution of perovskite,^[^ ^2^, ^4^, ^14^, ^53^ ^]^ facilitating a more comprehensive and intuitive understanding of the nucleation, crystallization and energy band evolution of perovskites.

## Conflict of Interest

The authors declare no conflict of interest.

## Supporting information

Supporting Information

Supplemental Video 1

Supplemental Video 2

Supplemental Video 3

Supplemental Video 4
