# Supplementary material for: Synchrotron Radiation‐Based In Situ GIWAXS for Metal Halide Perovskite Solution Spin‐Coating Fabrication
Source: Adv Sci (Weinh). 2024 Jul 11;11(35):2403778. doi: 10.1002/advs.202403778 (PMC11425288; doi:10.1002/advs.202403778)
Supplement: Supplementary file 1 — Supporting Information [file ADVS-11-2403778-s005.docx]

**Support information**

**Synchrotron radiation-based *in*** ***situ* GIWAXS for metal halide perovskite solution spin-coating fabrication**

Yingguo Yang^a,b,c^*, Shanglei Feng^b^, Xiaoxi Li^a,f^, Minchao Qin^d^, Lina Li^b^*, Xuyong Yang^e^, Renzhong Tai^b^*

*^a^School of Microelectronics, Fudan University, Shanghai 200433, China.*

*^b^Shanghai Synchrotron Radiation Facility (SSRF), Zhangjiang Lab, Shanghai Advanced Research Institute, Shanghai Institute of Applied Physics, Chinese Academy of Sciences, Shanghai 201204, China.*

*^c^State Key Laboratory of Photovoltaic Science and Technology, Fudan University, Shanghai 200433, China.*

*^d^Department of Physics, The Chinese University of Hong Kong, Shatin 999077, Hong Kong, China.*

*^e^Key Laboratory of Advanced Display and System Applications of Ministry of Education, Shanghai University; Shanghai 200072, China.*

*^f^Hangzhou Institute of Technology, Xidian University, Hangzhou, 311200, China*

^#^These authors (Yingguo Yang, Shanglei Feng and Xiaoxi Li) contribute equally to this paper.

^*^Correspondence and requests for materials should be addressed to yangyingguo@fudan.edu.cn, lilina@sinap.ac.cn(L.L.), tairenzhong@sinap.ac.cn(R. T.).

**Support Table and Figure**

*Table S1 In situ* GIWAXS experimental main parameters based on the SSRF[14-16], APS[9], CHESS[10, 11], and NSRRC[13] equipped with a spin-coater, sample chamber, and online glovebox.


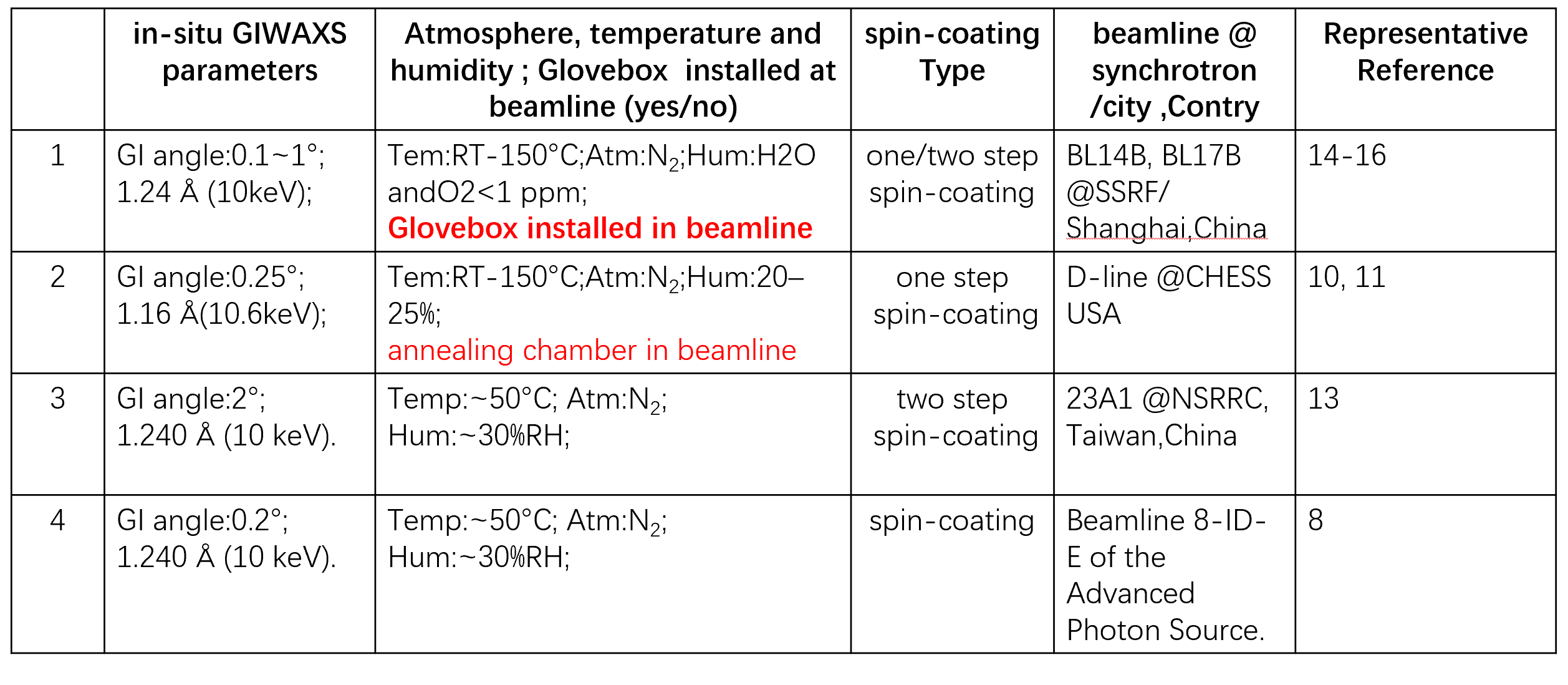


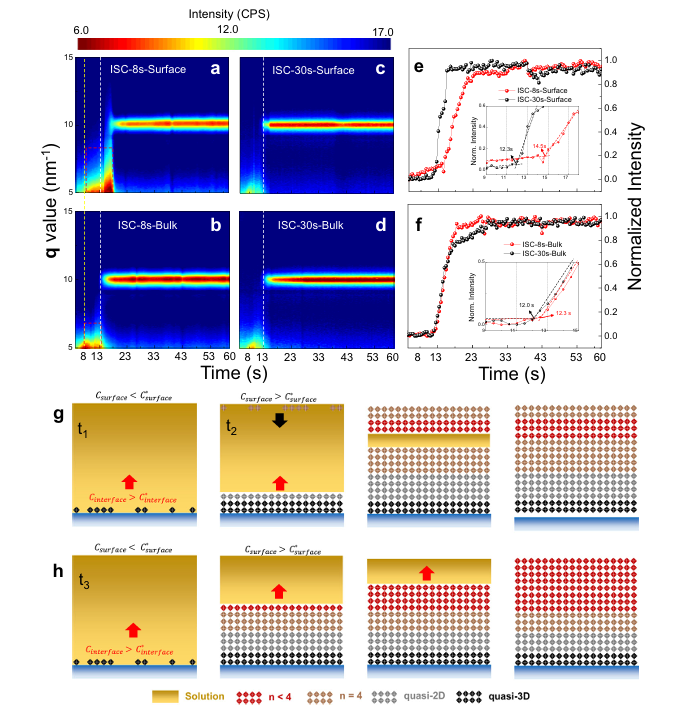


**Figure S1.** Crystallization comparison of thin-films fabricated with ISC and SC methods. Reproduced with permission. Copyright 2021, Springer Nature.^[15]^

**Reference:**

**[8]** Eric F. Manley, Joseph Strzalka, Thomas J. Fauvell, Nicholas E. Jackson,Matthew J. Leonardi, Nicholas D. Eastham, Tobin J. Marks, and Lin X. Chen,*In Situ* GIWAXS Analysis of Solvent and Additive Effects on PTB7 Thin Film Microstructure Evolution during Spin Coating. ***Adv. Mater.*** 2017, 1703933.

**[9]** Justin M. Hoffman, Joseph Strzalka, Nathan C. Flanders, Ido Hadar, Shelby A. Cuthriell,Qingteng Zhang, Richard D. Schaller, William R. Dichtel, Lin X. Chen, and Mercouri G. Kanatzidis. *In Situ* Grazing-Incidence Wide-Angle Scattering RevealsMechanisms for Phase Distribution and Disorientation in 2D Halide Perovskite Films, ***Adv. Mater.*** 2020, 2002812.

**[10]** Kai Wang, Ming-Chun Tang, Hoang X. Dang, Rahim Munir, Dounya Barrit,Michele De Bastiani, Erkan Aydin, Detlef-M. Smilgies, Stefaan De Wolf, and Aram Amassian. Kinetic Stabilization of the Sol–Gel State in Perovskites Enables Facile Processing of High-Efficiency Solar Cells,***Adv. Mater.*** 2019, 1808357.

**[11]** Y. H. Lou, et al. Seed-mediated superior organometal halide films by GeO_2_ nano-particles for high performance perovskite solar cells. Appl. Phys. Lett. 2016, 108, 053301.

**[12]** Minchao Qin, Kinfai Tse, Tsz-Ki Lau, Yuhao Li, Chun-Jen Su, Guang Yang, Jiehuan Chen,Junyi Zhu, U-Ser Jeng, Gang Li, Hongzheng Chen, and Xinhui Lu,Manipulating the Mixed-Perovskite Crystallization Pathway Unveiled by *In Situ* GIWAXS. ***Adv. Mater.*** 2019, 31, 1901284.

**[13] (a)** Minchao Qin, Haibo Xue, Hengkai Zhang, Hanlin Hu, Kuan Liu, Yuhao Li,Zhaotong Qin, Junjie Ma, Hepeng Zhu, Keyou Yan, Guojia Fang, Gang Li, U-Ser Jeng,Geert Brocks, Shuxia Tao, and Xinhui Lu. ***Adv. Mater.*** 2020, 2004630. (b)Minchao Qin, Pok Fung Chan, and Xinhui Lu, A Systematic Review of Metal Halide Perovskite Crystallization and Film Formation Mechanism Unveiled by *In Situ* GIWAXS, ***Adv. Mater.*** 2021, 33, 2105290.

**[14]** Yingguo Yang, Haizhou Lu, et al .Modulation of perovskite crystallization processes toward highly efficient and stable perovskite solar cells with MXene quantumdot-modified SnO_2_, ***Energy Environ. Sci.***, 2021, 14, 3447

**[15]** Yajie Yan, Yingguo Yang, Mingli Liang, Mohamed Abdellah, Tõnu Pullerits , Kaibo Zheng&Ziqi Liang,Implementing an intermittent spin-coating strategy to enable bottom-up crystallization in layered halide perovskites. ***Nature Comm***, 2021,12,6603.

**[16]** Liang Kuai, Junnan Li, Yajuan Li,Yusheng Wang, Pandeng Li, Yuanshuai Qin,Tao Song,Yingguo Yang, Zhuoying Chen, Xingyu Gao, and Baoquan Sun,Revealing Crystallization Dynamics and the Compositional Control Mechanism of 2D Perovskite Film Growth by *In Situ* Synchrotron radiation-based GIXRD. ***ACS Energy Lett.*** 2020, 5, 8−16
